# Trust in the Transplant Team Associated With the Level of Chronic Illness Management—A Secondary Data Analysis of the International BRIGHT Study

**DOI:** 10.3389/ti.2024.11704

**Published:** 2024-03-11

**Authors:** Juliane Mielke, Maan Isabella Cajita, Kris Denhaerynck, Sabine Valenta, Fabienne Dobbels, Cynthia L. Russell, Sabina De Geest, the BRIGHT study team

**Affiliations:** ^1^ Department of Public Health, Faculty of Medicine, Institute of Nursing Sciences, University of Basel, Basel, Switzerland; ^2^ College of Nursing, University of Illinois at Chicago, Chicago, IL, United States; ^3^ Practice Development and Research Division, Medical Directorate, University Hospital Basel, Basel, Switzerland; ^4^ Academic Center for Nursing and Midwifery, Department of Public Health and Primary Care, Faculty of Medicine, KU Leuven, Leuven, Belgium; ^5^ School of Nursing and Health Studies, Kansas City, MO, United States

**Keywords:** trust, chronic illness management, heart transplant, transplant team, behavioral outcomes

## Abstract

A trustful relationship between transplant patients and their transplant team (interpersonal trust) is essential in order to achieve positive health outcomes and behaviors. We aimed to 1) explore variability of trust in transplant teams; 2) explore the association between the level of chronic illness management and trust; 3) investigate the relationship of trust on behavioral outcomes. A secondary data analysis of the BRIGHT study (ID: NCT01608477; https://clinicaltrials.gov/ct2/show/NCT01608477?id=NCT01608477&rank=1) was conducted, including multicenter data from 36 heart transplant centers from 11 countries across four different continents. A total of 1,397 heart transplant recipients and 100 clinicians were enrolled. Trust significantly varied among the transplant centers. Higher levels of chronic illness management were significantly associated with greater trust in the transplant team (patients: AOR= 1.85, 95% CI = 1.47–2.33, *p* < 0.001; clinicians: AOR = 1.35, 95% CI = 1.07–1.71, *p* = 0.012). Consultation time significantly moderated the relationship between chronic illness management levels and trust only when clinicians spent ≥30 min with patients. Trust was significantly associated with better diet adherence (OR = 1.34, 95%CI = 1.01–1.77, *p* = 0.040). Findings indicate the relevance of trust and chronic illness management in the transplant ecosystem to achieve improved transplant outcomes. Thus, further investment in re-engineering of transplant follow-up toward chronic illness management, and sufficient time for consultations is required.

## Graphical Abstract

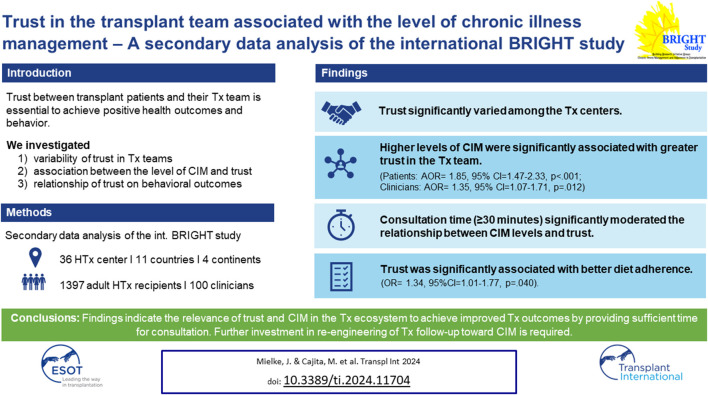

## Introduction

The importance of interpersonal trust (i.e., trust between patients and healthcare providers) in the healthcare context has been widely reported [1]. Trust occurs in vulnerable situations where an individual believes that another individual will act in their best interest [2]. This is especially true for chronically ill populations such as heart transplanted (HTx) patients. HTx patients face a high level of vulnerability due to potentially life-threatening complications and lifelong dependency on the HTx team providing follow-up care [3]. Trust has to be understood as a continuum, meaning that it is a complex and evolving phenomenon that can increase or decrease over time. Interpersonal trust relationships are supposed to positively affect patients’ attitudes, experiences (e.g., satisfaction with care [4–6]) and behavior (e.g., increased adherence to medication and treatment [6–8]). Further, trust is linked to patients’ health outcomes [2, 4, 6], health-related quality of life [4], and symptom-related outcomes [4].

Several factors are associated with higher interpersonal trust, and either relate to the patient (e.g., patients who are white, women, or older, or those with a better health status or a higher number of healthcare visits) or the physician (e.g., better communication skills, higher competence, or higher consultation time). In addition, service factors, e.g., the type of delivery system, continuity in care, and absence of economic or other pressures, affect patients’ trust in healthcare professionals [2, 7, 8].

**FIGURE 1:**
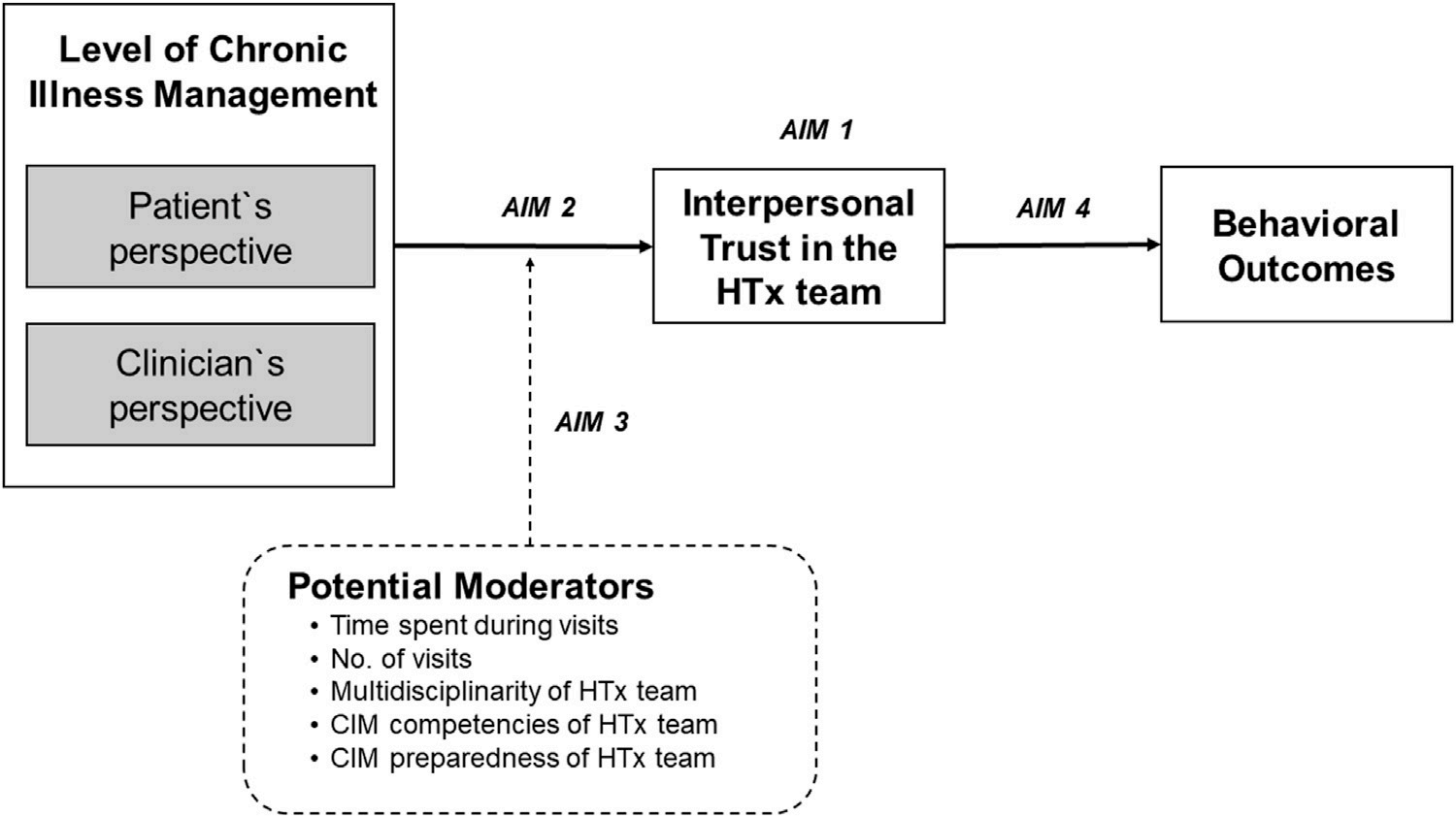
Conceptual model of studied variables and outcomes. Conceptualization of the Relationship between the Level of Chronic Illness Management (CIM) and Patients’ Interpersonal Trust in the Heart Transplant Team (HTx) on Health and Behavioral Outcomes.

While patient and clinician factors have been extensively examined, the relationship between trust and service outcomes—level of chronic illness management (CIM)—remain understudied [9]. Chronic illness management refers to a comprehensive and coordinated approach that focusses on optimizing the care provided to individuals living with long-term medical conditions. CIM programs based on the Chronic Care Model (CCM) [10] are designed to transform acute care driven health programs into patient centered integrated care and to address needs of the chronically ill, i.e., continuity of care, behavioral, self-management, and psychosocial support and patient participation [11]. The CCM is a framework that guides the development of care delivery models for the chronically ill to effectively improve patients’ clinical and behavioral outcomes and to enhance proactive patient and healthcare provider interactions [12]. Such interactions (e.g., during consultations) require interpersonal trust [13]. To assess, how well elements of the CCM have been implemented in a specific care program, the level of chronic illness management can be determined. CIM is a construct that can be assessed using validated instruments that allow patients and healthcare professionals to report how they perceive characteristics of clinical care processes [14, 15]. The higher the level of CIM, the more CCM elements were implemented. To our knowledge, there is no evidence on the association between CCM-based CIM programs and interpersonal trust, yet it is an important association with regards to teasing out a favorable ecosystem for HTx patients’ follow-up care, i.e., multilevel characteristics of care systems or processes that allow a CIM model of care to be implemented and sustained. Typically, HTx patients are cared for by an interdisciplinary HTx team across the transplant continuum in an HTx center with specific structural and care process characteristics. Studies that focus on interpersonal trust, however, do not consider the context in which these relationships occur. Therefore, this study aimed to 1) explore the variability of interpersonal trust in HTx teams among 36 HTx centers internationally; 2) explore whether the level of CIM of an HTx center is associated with trust in the HTx team; 3) investigate whether meso-level factors (e.g., time spent with the HTx team during follow-up) moderate the relationship between level of CIM and trust in the HTx team, and 4) investigate the relationship of trust in the HTx team on behavioral outcomes (Figure 1).

## Materials and Methods

### Design, Setting, and Sample

This study presents a secondary data analysis of the international, multicenter, cross-sectional Building Research Initiative Group: Chronic Illness Management and Adherence in Transplantation (BRIGHT) study (ID: NCT01608477; https://clinicaltrials.gov/ct2/show/NCT01608477?id=NCT01608477&rank=1). Detailed study procedures are described elsewhere [16, 17]. Briefly, using a multistage sampling approach of HTx centers, clinicians, and patients, CIM practice patterns and multilevel factors related to medication non-adherence were examined in 36 HTx centers from 11 countries across four continents (Europe, North America, South America, and Australia). A minimum of two HTx centers per country were included, if they had performed more than 50 HTx during the past 12–60 months. A convenience sample of 100 clinicians (1–5 per center) was chosen, using a random sample if more than five were eligible who had worked in the center for more than 6 months. Clinicians had to have spent more than 50% of their employment in direct clinical practice and have been familiar with the posttransplant outpatient care at the center. HTx patients (≥18 years of age) followed up in a participating center were included randomly if they were between 1–5 years post-transplant, first and single-organ transplant, able to read, understand and provide written informed consent. Data were collected between March 2012 and October 2015. The study was approved by the ethics committee of the University Hospital Leuven, Belgium and the ethics committees of each participating center. Written informed consent was obtained from all participating patients.

### Variables and Measurement

Data were collected from transplant directors, clinicians, and patients who completed a specific self-report BRIGHT questionnaire for each of these samples. In addition, patients’ sociodemographic data were collected during the enrollment interview and their clinical information was extracted from medical records (Table 1).

**TABLE 1:** Overview of variables and measurement tools.

Variable	Description (number of items, response options, scoring)	Instrument and psychometrics
Trust in the healthcare team	• 10 items measuring the level of interpersonal trust in the HTx-team covering the dimensions fidelity (caring and advocating for the patient’s welfare), competence, honesty, confidentiality, and global trust in the healthcare team	Adapted from the Wake Forest University Trust scale [18, 19]
	• 5-point Likert scale (1 = strongly disagree (low trust) to 5 = strongly agree (high trust))	
	• Higher average scores indicate more trust in the transplant team	
Level of chronic illness management (CIM)	Patient’s perspective	Short version of the Patient Assessment of Chronic Illness Care (PACIC) instrument [20, 21]; Cronbach`s α = .88 [17]
	• 11 items assessing practice patterns related to chronic illness management implemented in the HTx left	
	• 5-point Likert scale (1 = almost never to 5 = almost always)	
	• Total score ranging from 11 to 55 with higher scores indicating a higher degree of CIM	
	Clinician`s perspective	CIMI-BRIGHT [22]
	• 52 items covering the five building blocks of the Innovative Care for Chronic Conditions framework: 1) promote continuity and coordination (12 items); 2) encourage quality through leadership (7 items); 3) organize and equip healthcare teams (8 items); 4) support self-management (19 items); 5) use of information system (9 items)	Scale content validity = 0.86
	• 4-point Likert scale (1 = strongly disagree to 4 = strongly agree)	Interrater reliability (pilot tested): 75%–85% [22]; Cronbach`s α = .94 [17]
	• Average score with higher scores indicating higher level of CIM implemented	
Socio-demographic factors	6 items assessing the patient`s demographic profile (i.e., age, gender, race, country, educational level, employment status)	BRIGHT patient interview questionnaire [17]
Clinical information	2 items assessing patient`s clinical profile, i.e., the number of years post HTx, comorbidities and the number of rejections experienced (expressed as the number of treated rejections until time of study enrolment, divided by the years in post-transplant follow-up in years)	Medical record
Potential meso-level moderators		
Time spent with patients during follow-up visits	Patient`s perspective on time spent with all members of the HTx team during one follow-up visit	BRIGHT patient self-report (written) questionnaire [17]
	• 1 item	
	• five choices (<5 min, 5–10 min, 11–20 min, 21–30 min, >30 min)	
	Clinician`s perspective on time spent with each patient during one follow-up visit	BRIGHT clinician questionnaire [17]
	• 1 item	
	• Average total time per patient (in minutes)	
Number of visits	3 items assessing the total number of visits scheduled for patients within the first month, first 6 months, 1 year and 3 years	BRIGHT transplant director questionnaire [17]
Multidisciplinarity of the HTx team	• 1 item assessing multidisciplinarity of the HTx team, i.e., HTx team is composed of physician (s), nurse (s), and at least one other type of healthcare professional (either a social worker, psychiatrist, psychologist, pharmacist, dietitian, physical therapist, or occupational therapist)	BRIGHT transplant director questionnaire [17]
	• Dichotomous answer format (yes/no)	
Chronic illness management competencies	• 24 items assessing CIM competencies of the HTx team including 1) patient-lefted care (7 items); 2) partnering (2 items); 3) quality improvement (8 items); 4) information and communication technology (4 items); 5) public health perspective (3 items)	BRIGHT clinician questionnaire Cronbach`s α = .96 [17]
	• 4-point Likert scale (1 = strongly disagree to 4 = strongly agree; 5 = don`t know; set to missing)	
	• Average score with higher cores reflecting higher degree of core competencies	
Chronic illness management level of preparedness	• 5 items assessing level of preparedness in view of the skills and availability of equipment or tools to facilitate chronic care	BRIGHT clinician questionnaire Cronbach`s α = .82 [17]
	• 4-point Likert scale (1 = strongly disagree to 4 = strongly agree; 5 = don`t know; set to missing)	
	• Average score with higher cores reflecting higher degree of preparedness	
***Behavioral Outcomes***		
Physical activity	• 2 items asking if a patient was sufficiently active (yes/no)	2-item Brief Physical Activity Assessment tool [23]
	• Sufficiently active was defined as ≥ 3x/week 20 min of vigorous and/or ≥ 5x/week 30 min of moderate physical activity	Criterion validity of self-report against electronic monitoring gold standard measurement: κ statistic 0.14–0.40 [23]
Dietary adherence	• 5 items measuring patient`s adherence to low salt, low calorie, low saturated fat, low sugar or other kind of dietary guidelines	BRIGHT patient self-report (written) questionnaire [17]
	• 5-point Likert scale (1 = never to 5 = always)	
	• Adherent were those who were prescribed a diet, scoring often/always to any of the 5 diets	
Sun protection	• 4 items assessing consistency of sun protection	Swiss study on health of people with cancer, leukemia, tumor in childhood (Swiss Childhood Cancer Registry) [24] and Cambridge University Hospitals’ perception of skin cancer in transplant recipients scale [25]; Cronbach`s α = .59 [17]
	• 5-point Likert scale (1 = never to 5 = always)	
	• Average score	
Smoking status	1 item assessing smoking status, i.e., currently smoking, stopped less than 1 year ago, stopped more than 1 year ago, or never smoked	Item from Swiss Health survey (Swiss Federal Statistical Office 2008) [26]
Alcohol intake	• 2 items measuring the level of alcohol intake, i.e., whether the patients used alcohol (yes/no), and in case they did, how many alcoholic drinks were consumed per week	BRIGHT patient self-report (written) questionnaire [17]
	• Categorization into non-drinkers; moderate drinkers (1 drink/day for women, 2 drinks/day for men), or heavy drinkers (>1 drink/day for women, >2 drinks/day for men) [27]	
Language congruency	• 1 item measuring if the HTx team communicated in the patient`s mother tongue or in a language they mastered fluently (either via an interpreter or directly)	BRIGHT patient interview questionnaire [17]
	• Dichotomous answer format (yes/no)	
Health literacy	• 1 item rating patient`s confidence in filling out medical forms	Subjective Health Literacy Screener [28]
	• 5-point Likert scale (1 = none of the time to 5 = all of the time)	Concurrent validity: with the Short Test of Functional Health Literacy in (AUC = .72-.74; with the Rapid Estimate of Adult Literacy in Medicine (AUC = .81-.84) [29]
	• Dichotomized into adequate (≥4) and inadequate (≤3) health literacy [28]	
Number of comorbidities	• 19 items assessing post-HTx comorbidity	Adapted Charlson Comorbidity Index [30]
	• Dichotomous answer format (yes/no)	

Note. Abbreviations: CIM, chronic illness management; HTx, heart transplantation.

#### Main Outcomes

*Trust in the healthcare team* was part of the patient questionnaire and adapted from the 10-item Wake Forest University Trust scale measuring the level of interpersonal trust, i.e., fidelity (caring and advocating for the patient’s welfare), competence, honesty, confidentiality, and global trust in the healthcare team [18]. The three negatively worded items were recoded and an average score was calculated for each patient-participant with a higher overall score (range 1–5) indicating higher trust. Given that the trust variable was not normally distributed, it was dichotomized using the median score for the patient-sample for easier interpretation of interaction terms. Sensitivity analysis was performed using tertiles instead of the median with similar results.

The *level of CIM implemented* in the HTx program was measured from two perspectives. First, patient-participants completed the 11-item short version of the Patient Assessment of Chronic Illness Care (PACIC) instrument [20]. This instrument measures specific actions or qualities of care in the delivery system, which are congruent with the CCM and were observed over a recall period of 6 months. The items were aggregated for each patient-participant, with the total score ranging from 11 to 55. Higher scores indicate a higher degree of CIM. The median score of the patient-sample was used to dichotomize the PACIC variable. Second, implementation of CIM was measured from the clinician’s perspective by applying the investigator-developed CIMI-BRIGHT clinician questionnaire (The Chronic Illness Management Implementation—Building Research Initiative Group: Chronic Illness Management and Adherence in Transplantation (CIMI-BRIGHT) instrument), which consists of 52 items covering the five building blocks of the Innovative Care for Chronic Conditions framework [22]. An average score was calculated for each clinician-participant and then the median score for the clinician-sample was used to dichotomize the CIMI-BRIGHT variable.

#### Potential Meso-Level Moderators

*Time spent with patients during follow-up visits* was assessed from two perspectives. Patient-participants were asked how much time all members of their HTx team spend with them on regular follow-up visits. Each participating clinician was asked for the average total time (in minutes) they spend with each patient at the outpatient HTx clinic. Both *time* variables were then dichotomized using 20 min and 30 min as the cut-off points—these time points were chosen given the distribution of the continuous clinician-time variable and how it aligned with the ordinal patient time-variable.

The typical *number of visits* within the first month, first 6 months, 1 year, and 3 years were extracted from the transplant director’s BRIGHT questionnaire. Similarly, information regarding the *multidisciplinarity* of the HTx team was collected from the director’s questionnaire. The *CIM competencies* of HTx team and *CIM level of preparedness* of healthcare workers were assessed using the investigator-developed clinician questionnaire including 24 and five items, respectively. Scores were averaged, with higher scores reflecting a higher degree of core competencies and preparedness.

#### Behavioral Outcomes

The patient questionnaire also included five health behaviors: *Physical activity* was measured by the 2-item Brief Physical Activity Assessment tool [23], asking if a patient was sufficiently active. *Dietary adherence* recorded patient’s self-reported adherence, as applicable, to low salt, low calorie, low saturated fat, low sugar, or other kind of dietary guidelines. *Sun protection* was measured using 4 items assessing consistency of protection against the sun [24, 25]. *Smoking status* was based on whether patients were currently smoking, stopped less than 1 year ago, stopped more than 1 year ago, or never smoked [26]. *Alcohol intake* assessed the level of alcohol consumption by two items i.e., whether the patient used alcohol, and in case they did, how many alcoholic drinks were consumed per week. They were then categorized into non-drinkers; moderate drinkers, or heavy drinkers [27].

*Language congruency* was measured by asking patients during the interview if the HTx team communicated in their mother tongue or in a language they mastered fluently (either via an interpreter or directly). *Health literacy* was assessed as part of the written questionnaire by rating confidence in filling out medical forms, using a 5-point scale (1 = none of the time to 5 = all of the time) and then dichotomized into adequate (≥4) and inadequate (≤3) health literacy [28]. Lastly, *number of comorbidities* post-HTx was assessed using an adapted Charlson Comorbidity Index [30].

### Statistical Analysis

Descriptive statistics were calculated for all study variables. The Kruskal-Wallis test was used to examine whether there were differences in trust in the HTx team across the 36 HTx centers. Whether level of CIM was associated with trust in the HTx team was examined using simple and multiple logistic regression. Meanwhile, moderation analysis was performed to determine whether meso-level factors affected the direction and/or strength of the relationship between level of CIM and trust. To examine whether trust could predict behavioral outcomes, simple and multiple logistic regressions were performed, whereby the multiple models were equally controlled for potential confounders that were statistically significant. Finally, marginal effects were calculated to better communicate the practical significance of the findings [31]. Analyses were conducted in Stata v16.1.

## Results

Characteristics of the participants are shown in Table 2. The proportion of physicians and nurses included reflected the composition of HTx teams in clinical practice. Less than 2% of the data were missing; hence, no imputation was performed.

**TABLE 2:** Patients’ demographic and clinical characteristics.

Variable	
Patients	
Age^a^ (*n* = 1,379)	53.5 ± 13.3 years
Gender^b^ (*n* = 1,390)	
Male	1,011 (72.7)
Female	379 (27.3)
Race/Ethnicity^b^ (*n* = 1,381)	
White	1,186 (85.9)
Black	80 (5.8)
Asian	27 (1.9)
Hispanic	29 (2.1)
Other	59 (4.3)
Country^b^ ^,^ ^c^ (*n* = 1,379)	
Belgium	74 (5.3)
Spain	227 (16.2)
France	160 (11.5)
Canada	121 (8.7)
USA	340 (24.3)
Australia	51 (3.7)
Italy	111 (7.9)
United Kingdom	99 (7.1)
Germany	67 (4.8)
Switzerland	47 (3.4)
Brazil	100 (7.2)
Educational Attainment^b^ (*n* = 1,377)	
Primary School	187 (13.6)
Secondary School	426 (30.9)
University	764 (55.5)
Employment Status^b^ (*n* = 1,391)	
Employed	413 (29.7)
Unemployed	978 (70.3)
Years post HTx^a^ (*n* = 1,378)	3.37 ± 1.4 years
Health Literacy^b^ (*n* = 1,364)	
Adequate	912 (66.9)
Inadequate	452 (33.1)
Language Congruency^b^ (*n* = 1,390)	
Spoke different languages	15 (1.1)
Spoke the same language	1,375 (98.9)
Number of Comorbidities^a^ (*n* = 1,394)	1.44 ± 1.6
Trust in the healthcare team^a^ (*n* = 1,378)	4.59 ± 0.49
Level of CIM^a^ (PACIC)	38.32 ± 10.9^d^
Time spent with clinicians^b^ (*n* = 1,374)	
<5 min	8 (0.57%)
5–10 min	68 (4.87%)
11–20 min	382 (27.34%)
21–30 min	388 (27.77%)
>30 min	528 (37.8%)
Clinicians	
Age^a^ (*n* = 98)	45.83 ± 10.2
Gender^b^ (*n* = 99)	
Male	12 (12.1%)
Female	87 (87.9%)
Profession^b^ (*n* = 100)	
Nurse	90 (90%)
Physician	3 (3%)
Other	7 (7%)
Years practicing in Tx^a^ (*n* = 99)	11.9 ± 7.7
Level of CIM^a^ (CIMI-BRIGHT)	2.9 ± 0.27^e^
Time spent with patients in minutes^a^ (*n* = 94)	36.8 ± 34.0

Note: Abbreviations: HTx, heart transplantation.

^a^ Values given are mean ± SD.

^b^ Values given are n (%).

^c^ Country where the center is located.

^d^ Total scores ranging from 11 to 55 with higher scores indicating a higher degree of CIM.

^e^ Average scores ranging from 1 to 4 with higher scores indicating higher level of CIM, implemented.

There was significant variability in the level of CIM [PACIC: chi-square (35 df, N = 36) = 209.3, *p* < 0.001; CIMI: chi-square (35 df, N = 36) = 1,396, *p* < 0.001] and trust in the healthcare team [chi-square (35 df, N = 36) = 221.5, *p* < 0.001] among the 36 HTx centers (Supplementary Material). HTx recipients who indicated that they had received higher levels of CIM were more likely to have greater trust in the HTx team. This finding was consistent whether level of CIM was measured from the patient’s perspective (adjusted odds ratio [AOR] = 1.85, 95% CI = 1.47 to 2.33, *p* < 0.001) or from the clinician’s perspective (AOR = 1.35, 95% CI = 1.07 to 1.71, *p* = 0.012), and even after controlling for potential confounders (age, gender, race, education level, employment status, number of years post HTx, health literacy, language congruency, and comorbidities) (Table 3). However, when controlling for the country where the HTx center was located, the level of CIM from clinicians was no longer significant (AOR = 0.94, 95% CI = 0.67 to 1.30, *p* = 0.703). Using USA as reference group, HTx patients from France, Germany, and Switzerland had lower odds of having high trust (AOR = 0.16–0.46), while HTx patients from Canada and the UK had higher odds of having higher trust (AOR = 1.86). Meanwhile, education became significant (*p* = 0.002- and *p* = 0.017), indicating that patients with higher education had greater odds of having higher trust (AOR = 1.67–1.96). The calculated marginal effects showed that an average HTx recipient who received lower levels of CIM had a 42.4% probability of trusting their HTx team. Meanwhile, a comparable HTx recipient who received higher levels of CIM had a 57.7% probability of trusting their HTx team.

**TABLE 3:** Associations between chronic illness management level and trust.

Independent variables	Unadjusted bivariate analysis		Adjusted multivariate regression analysis		
	OR	95% CI	OR	95% CI	*p*-value
PACIC^a^	1.91	1.54–2.36	2.08	1.62–2.65	<0.001
CIMI-BRIGHT^b^	1.33	1.08–1.65	0.94	0.67–1.30	0.703
*Age*			*1.02*	*1.00–1.03*	*<0.001*
*Gender*			*1.20*	*0.92–1.56*	*0.184*
*Race*					
* Black*			0.93	0.55–1.57	0.786
* Asian*			0.78	0.32–1.90	0.587
* Hispanic*			0.50	0.21–1.17	0.109
* Other*			0.79	0.43–1.47	0.462
*Education*					
* High school*			1.67	1.10–2.54	0.017
* College*			1.96	1.27–3.02	0.002
*Employment*			1.09	0.84–1.42	0.497
*Country*					
* Belgium*			1.00	0.58–1.75	0.988
* Spain*			0.96	0.60–1.54	0.861
* France*			0.46	0.28–0.77	0.003
* Canada*			1.86	1.13–3.07	0.015
* Australia*			0.82	0.39–1.73	0.598
* Italy*			1.62	0.95–2.75	0.074
* United Kingdom*			1.86	1.12–3.08	0.017
* Germany*			0.16	0.08–0.33	<0.001
* Switzerland*			0.23	0.10–0.51	<0.001
* Brazil*			1.46	0.85–2.49	0.166
*Years post-HTx*			0.94	0.86–1.02	0.141
*Health literacy*			1.58	1.23–2.03	<0.001
*Language congruence*			1.77	0.49–6.35	0.381
*Comorbidities*			1.03	0.96–1.11	0.372

Note. Abbreviations: CI, confidence interval; OR, odds ratio.

^a^ Short version of the Patient Assessment of Chronic Illness Care (PACIC) instrument [20, 21].

^b^ The Chronic Illness Management Implementation—Building Research Initiative Group: Chronic Illness Management and Adherence in Transplantation (CIMI-BRIGHT) instrument [22].

Reference groups: race (White), education (primary school), country (United States).

Among the potential moderators, only time spent with the patients during follow-up visits was significant, i.e., the association between CIM and trust was stronger when consultation time was ≥30 min. This moderation effect was only present when consultation time was >20 min, measured from both the patient’s (OR = 1.61, 95% CI 1.03 to 2.53, *p* = 0.037) and from the clinician’s perspective (OR = 1.56, 95% CI 1.00 to 2.42, *p* = 0.048).

Results of the bivariate and multiple logistic regressions are presented in Table 4. Bivariate analyses showed that trust in the HTx team was significantly associated with smoking and diet adherence. Wherein patients, who had greater trust in their HTx team, were less likely to smoke (OR = .46, 95% CI .25 to .84, *p* = 0.012) and more likely to adhere to their recommended diets (OR = 1.40, 95% CI 1.08 to 1.82, *p* = 0.012). However, after controlling for age, gender, race, education, employment status, years post HTx, health literacy, and comorbidities, only the relationship between trust and diet adherence remained significant (OR = 1.34, 95%CI 1.01 to 1.77, *p* = 0.040). The calculated marginal effects showed that an average HTx recipient who highly trusts their HTx team (i.e., Trust score = 5) is 2.5 times more likely to adhere to their recommended diet compared to an average HTx recipient who has low trust towards their HTx team (i.e., trust score = 1).

**TABLE 4:** Associations between trust and health outcomes.

	Outcome variables	Unadjusted bivariate analysis		Adjusted multilevel regression analysis	
		OR	95% CI	OR	95% CI
IV: Trust	Physical activity	0.95	0.71 to 1.28		
	Diet adherence	1.40^a^	1.08 to 1.82	1.34^a^	1.01 to 1.77
	Sun protection	1.27	.96 to 1.69		
	Smoking	0.46	0.25 to 0.84	0.59	0.31 to 1.11
	Alcohol intake	0.75	0.53 to 1.06		

Note. Abbreviations: CI, confidence interval; OR, odds ratio.

^a^ *P* < 0.05; For aim 4, trust was treated as the predictor variable and the health outcomes were the response variables. The final model was adjusted for age, gender, race, education, employment status, years post HTx, health literacy, and comorbidities.

## Discussion

In this study, we observed significant variability in trust in HTx team across the 36 HTx centers. Additionally, associations of CIM, trust in the HTx team and one patient behavioral outcome in HTx follow-up were identified.

First, higher levels of CIM were associated with greater trust in the HTx team, even after adjusting for potential confounders. However, when we controlled for country, the level of CIM from the clinician’s perspective was no longer significant, indicating that the association between clinician-reported CIM levels and trust may be contingent upon the country context. Although country may not serve as an ideal indicator of social and cultural disparities, it is posited to be a more suitable indicator compared to race. Previous studies only focused on patient-level aspects of CIM, e.g., continuity of care [2, 7, 8] or physicians` communication skills [2, 7], were positively associated with greater trust in individual healthcare professionals. Yet, the strength of our study is having examined CIM meso-level factors with validated measurement tools from both the patient and clinician perspectives, resulting in consistent findings in each case.

Also, visit duration has been described as important for establishing interpersonal trust [8, 32]. In Fiscella et al.’s [32] study, visit duration independently predicted trust (0.05 SD, 95%CI 0.03–0.06). Patients’ trust in their primary care physician increased by every minute increase in visit duration (0.01 SD, 95% CI 0.001–0.02) [32]. However, in our study, a stronger association between the level of CIM and trust was found when visit duration was ≥30 min. Indeed, given the complexity of HTx follow-up care and its importance on patients’ health outcomes, it seems reasonable that HTx patients require more time for follow-up than patients in primary care settings. In addition, our findings shed light on the “dose” of time needed during consultations. Yet, further research on aspects contributing to trust during consultation is required.

In fact, the positive association of CIM and trust seems not surprising, when considering relevant components of CCM based CIM programs [10]. Largely overlapping with aspects increasing interpersonal trust, those components include availability of standards and training for clinicians (e.g., communication), patient-centered care, i.e., well informed and activated patients making their own choices, as well as care coordination of and advocacy for patients [33]. Another relevant aspect of CIM and driver of health outcomes include healthcare teams’ multidisciplinarity in HTx follow-up. In the BRIGHT study, the majority of included transplant centers (80.6%) involve multidisciplinary teams in HTx follow-up with no significant variability in the type of professionals within the HTx teams across HTx centers [34]. However, larger, multidisciplinary teams run the risk of individual healthcare providers working in silos and responsibilities for a patient not being clearly defined. To enable trust in multidisciplinary teams, care concepts based on CCM are needed in HTx centers to ensure, for example, continuity in care of the patient and support for self-management [35].

Second, we found trust significantly independently associated with diet adherence, even after controlling for potential confounders. In general, the association of trust in healthcare professionals and behavioral outcomes such as adherence (medication, exercise, diet), self-care activities, preventive care (r = 0.14, 95% CI 0.10–0.19, *p* < 0.001) was already described in Birkhäuer et al.’s [4] meta-analysis on 21 studies including a total of 26′642 patients. Further studies highlighted a positive influence of interpersonal trust on following physicians` recommendations (e.g., diet, lifestyle) [5, 8], use of services (e.g., screening) [6, 8] and adhering to medication and treatment [2, 6–8]. However, these studies only focused on trust in individual professionals, whereas our study takes a broader perspective and focusses on trust in the HTx team, reflecting current HTx practice. Our findings indicate CIM, trust and patient outcomes are closely related. While only one behavioral outcome was significantly associated with trust in our multivariate analysis, CIM itself can have a positive effect on behavioral and health outcomes (e.g., patient survival one-year post-Tx) [15]. Further, studies in renal Tx research show associations of CIM with increased medication adherence [36], improved quality of life [36], fewer emergency room visits [37], fewer hospital admissions [37, 38] and reduced mortality [38]. To enhance HTx patients’ behavioral and health outcomes, a systems perspective is needed, with not only focusing on interventions at patient-level, but also at re-engineering care processes in HTx follow-up towards CIM. This includes leadership accounting for trust as an important factor in HTx care, development of standards, best practices and training (e.g., communication and relationships skills) for the multidisciplinary HTx team, measuring, monitoring and reporting patient trust [33]. Further measures relevant to increasing patient’s trust in their HTx team include working towards an ecosystem that provides continuity of care and care coordination and allows patient centeredness and shared decision making within a CIM model [33, 39, 40]. The SMILe care model (Integrated Care Model (ICM) for SteM cell transplantatIon faciLitated by eHealth), for example, is one such care model that could potentially serve as a blueprint also for the care of HTx patients. Based on CIM building blocks, the SMILe-ICM aims to reengineer follow-up care of allogeneic stem cell transplanted patients and consists of four intervention modules to support patient self-management and health behaviors (i.e., monitoring & follow-up of vital signs, symptoms and health behavior; infection prevention; physical activity; medication adherence) [41–44].

However, the successful and sustainable implementation of complex interventions based on CIM principles and supporting trust into clinical practice is challenging due to healthcare, organizational, social, economic, and policy related barriers, among others [35, 45]. Implementation science supports the uptake of such interventions into routine practice and thus improves both health care services’ quality and effectiveness [46]. Further, core and adaptable components of complex interventions can be adapted and fitted to the local context in which they will be delivered. Key implementation science elements supporting a shift towards CCM entail contextual analysis, stakeholder involvement, the use of strategies supporting implementation as well as research designs focusing on both implementation and effectiveness outcomes (i.e., hybrid designs) [47].

### Limitations

Our study has several limitations. First, the cross-sectional study design does not allow causal inferences to be drawn. Second, a longitudinal analysis of trust over time could not be performed. Trust has to be understood as a continuum and may change over time. Since HTx patients usually receive life-long follow-up, changes in interpersonal trust relationships could point to aspects of CIM that are specifically relevant for patients’ trust throughout the transplant continuum. Those specific measures could be taken to support trust relationships in practice over time. Third, most data analyzed in this study rely on self-reports from patients and clinicians, introducing a potential for inaccuracies, which could be mitigated by incorporating routine data, for example. Fourth, since we included Tx survivors beyond one-year post-Tx, outcome events in the first year were not considered. These outcomes should be also included in further studies. Further, the fact that 86% of the patients were white limits the assessment of social and cultural differences in perceptions of interpersonal trust. Fifth, the majority of clinicians involved in this study (90%) were nurses. Nurses and other transplant clinicians might differ in their evaluation on the level of chronic illness management as nurses are typically more involved in patient self-management and also typically have a higher sensitivity of psychological issues. Finally, given the limitation due to using secondary data, we did not assess the link of trust on clinical outcomes moderated by service outcomes. Moreover, other potentially important factors such as use of eHealth, distance from Tx-center, health outcomes (e.g., acute rejection, survival) or emotional moderators such as the patient`s mental health concerns could not be examined given the nature of this study.

### Conclusion

To our knowledge, this is the first study linking CIM and interpersonal trust to service-level outcomes. We observed significant associations between CIM levels and trust in the HTx team moderated by consultation time, and a significant association between trust and diet adherence. Our findings highlight the need to consider trust and CIM in the HTx follow-up ecosystem as important factors as a basis for optimal transplant outcomes. Thus, further investment in re-engineering of HTx follow-up toward CIM, as well as allowing sufficient time for consultations, is required. Using longitudinal study designs, further research should focus on changes in trust over the transplant continuum and its influences on behavioral and clinical outcomes.

## Data Availability

Original datasets are not openly available due to reasons of privacy and are available from the corresponding author upon reasonable request.

## The BRIGHT Study Team

The BRIGHT Study Team consists of Lut Berben (Institute of Nursing Science, University of Basel, Switzerland); Marisa G. Crespo-Leiro (Complexo Hospitalario Universitario A Coruña (CHUAC), CIBERCV, INIBIC, Universidade da Coruña (UDC), La Coruña, Spain); Sandra Cupples (U.S. Department of Veterans Affairs, Veterans Health Administration, USA); Patricia M. Davidson (School of Nursing, The Johns Hopkins University, Baltimore, Maryland, USA); Paolo De Simone (Azienda Ospedaliero-Universitaria Pisana, Ospedale Cisanello, Pisa, Italy); Albert Groenewoud (Astellas Pharma Europe Ltd., UK); Christiane Kugler (Hannover Medical School, Germany); Linda Ohler (George Washington University, USA); Johan Van Cleemput (University Hospitals Leuven, Belgium); Alain Jean Poncelet (Cliniques Universitaires Saint-Luc, Brussels, Belgium); Laurent Sebbag (Hôpital Louis Pradel, Lyon, France); Magali Michel (Hôpital Nord Laennec, Nantes, France); Andrée Bernard (Hôpital Universitaire Pitié-Salpêtrière, Paris, France); Andreas Doesch (University Hospital Heidelberg, Germany; Asklepios Hospital Bad Salzungen, Bad Salzungen, Germany); Ugolino Livi (University Hospital Udine, Italy); Luciano Potenta (University of Bologna, Italy); Vicens Brossa-Loidi (Hospital de Sant Pau, Barcelona, Spain); Javier Segovia-Cubero (Hospital Puerta de Hierro, Madrid, Spain); Luis Almenar-Bonet (Hospital Universitari i Politècnic La Fe de Valencia, Spain); Carmen Segura Saint-Gerons (Hospital Univeritario Reina Sofia, Córdoba, Spain); Paul Mohacsi (University Hospital of Bern, Switzerland); Eva Horvath (University Hospital Zurich, Switzerland); Cheryl Riotto (Papworth Hospital, Cambridge, UK); Gareth Parry (Freeman Hospital, Newcastle, UK); Ashi Firouzi (Royal Brompton & Harefield NHS Foundation Trust, London, UK); Stella Kozuszko (Toronto General Hospital, Canada); Haissam Haddad (University of Ottawa Heart Institute, Canada); Annemarie Kaan (St Paul’s Hospital, Vancouver, Canada); Grant Fisher (London Health Sciences Centre, Ontario, Canada); Tara Miller (Duke University Hospital, North Carolina, USA); Maureen Flattery (Virginia Commonwealth University Health System, USA); Kristin Ludrosky (Cleveland Clinic, Ohio, USA); Bernice Coleman (Cedars-Sinai Medical Center, California, USA); Jacqueline Trammell (Kaiser Permanente Santa Clara Medical Center, California, USA); Flavio R. Epstein (Kaiser Permanente Santa Clara Medical Center, California, USA); Katherine St. Clair (St Luke’s Hospital, Missouri, USA); Andrew Kao (St Luke’s Hospital, Missouri, USA); Maria Molina (Hospital of the University of Pennsylvania, USA); Karyn Ryan Canales (Ochsner Medical Center, New Orleans, Louisiana, USA); Samira Scalso de Almeida (Hospital Israelita Albert Einstein, São Paulo & Hospit.al Municipal Vila Santa Catarina - Ministerio da Saude PROAD/-SUS, Sao Paulo, Brazil); Bartira de Aguiar Roza (Paulista School of Nursing, Federal University of Sao Paulo, Sao Paolo, Brazil); Andrea Cotait Ayoub (Instituto Dante Pazzanese de Cardiologia, São Paulo, Brazil); Fernanda Barone (Instituto do Coração da Universidade de São Paulo, Brazil); Michelle Harkess (St Vincent’s Hospital, Sydney, Australia); Joanne Maddicks-Law (The Prince Charles Hospital, Brisbane, Australia).

## Abbreviations

AOR, adjusted odds ratio; BRIGHT, Building Research Initiative Group: Chronic Illness Management and Adherence in Transplantation (BRIGHT) study; CCM, Chronic Care Model; CIM, chronic illness management; CIMI-BRIGHT, The Chronic Illness Management Implementation—Building Research Initiative Group: Chronic Illness Management and Adherence in Transplantation (CIMI-BRIGHT) instrument; HTx, heart transplantation; PACIC, Patient Assessment of Chronic Illness Care (PACIC) instrument.
