# Supplementary material for: Trust in the Transplant Team Associated With the Level of Chronic Illness Management—A Secondary Data Analysis of the International BRIGHT Study
Source: Transpl Int. 2024 Mar 11;37:11704. doi: 10.3389/ti.2024.11704 (PMC10961910; doi:10.3389/ti.2024.11704)
Supplement: Supplementary file 1 [file DataSheet1.docx]

**Supplementary Material**

**Figure S1** Variability in Chronic Illness Management (CIM) Level among Transplant Centers (Patient Perspective)

Kruskal Wallis test: chi-squared = 209.134 with 35 d.f. | probability = 0.0001; chi-squared with ties = 209.336 with 35 d.f. | probability = 0.0001; Interpretation: The variability in the PACIC scores is statistically significant.

**Figure S2** Variability in Chronic Illness Management (CIM) Level among Transplant Centers (Clinician Perspective)

Kruskal Wallis test: chi-squared = 1394.478 with 35 d.f. | probability = 0.0001; chi-squared with ties = 1396.000 with 35 d.f. | probability = 0.0001; Interpretation: The variability in the CIMI-BRIGHT scores is statistically significant.

**Figure S3** Variability in Patient`s interpersonal Trust in the Heart Transplant Team (HTx) among Transplant Centers

Kruskal Wallis test: chi-squared = 210.926 with 35 d.f. | probability = 0.0001; chi-squared with ties = 221.472 with 35 d.f. | probability = 0.0001; Interpretation: The variability in the Trust scores is statistically significant.
